# Current Espghan Guidelines for Celiac Disease in Pediatric Age, Tertiary Care Center Experience: A Proposal for Further Simplification

**Published:** 2019-01-12

**Authors:** M Malamisura, R Colantuono, V.M Salvati, R Croce, G D’Adamo, T Passaro, E D’Angelo, M Boffardi, A Garzi, B Malamisura

**Affiliations:** 1 Academic Department of Pediatrics, Ospedale Pediatrico Bambino Gesù, IRCCS, University of Rome, Italy; 2 Post-graduate School of Pediatrics – University of Salerno, Italy; 3 Pediatric Unit and Center for Celiac Disease – University Hospital of Salerno, Campus of Cava de’ Tirreni, Italy; 4 Mini-invasive and Robotic Pediatric Surgery – Maternal and Child Department, University of Salerno, Italy

**Keywords:** celiac disease, duodenal biopsy, anti-tissue transglutaminase, haplotypes

## Abstract

According to the 2012 ESPGHAN criteria for diagnosis of celiac disease (CD), duodenal biopsy (DB) can be avoided in children with a clear malabsorption syndrome, anti-tissue transglutaminase IgA (tTG2) ≥ 10x the cut-off, anti-endomysium IgA (EMA) and HLA DQ2/DQ8 genes. The aim of this study is to report our experience and evaluate the accuracy of the actual guidelines.

**Patients and methods:**

This is a retrospective study conducted on all patients diagnosed CD from 2012 to 2018 in our Center. For all patients enrolled were analyzed: data of family history, symptoms, serology, genetics, Marsh grade and follow-up.

**Results:**

A total of 481 children [mean age 6,4 yrs; F:M= 1.8:1] were included in the study. The mean age of patients who were not subject to DB was lower (4.51 yrs) comparing with patients that received DB (6.48 yrs). Out of the 256 patients with anti-tTG2 ≥ 10 fold, 121 underwent DB because of mild symptoms (84/121) or no symptoms (37/121). In all cases Marsh type 3 was found and HLA haplotypes was compatible with CD diagnosis.

**Conclusions:**

Our study confirms that the serology has a primary importance to diagnose CD, regardless of the symptoms. These data suggest that biopsy and HLA haplotypes search, in presence of anti-tTG2 IgA ≥ 10x the cut-off, are wasteful and unhelpful for the patients.

## I. INTRODUCTION

Celiac Disease (CD) is defined as a systemic immune-mediated disorder responsible for a permanent inflammatory enteropathy, caused by the ingestion of proteins contained in some cereals (gliadin in wheat, hordein in barley and secalin in rye) that are called prolamines, in people with specific human class II leukocyte antigen (HLA haplotype DQ2 and/or DQ8).

CD is characterized by variable clinical manifestations that may include intestinal and extraintestinal signs and symptoms as well as by high titers of specific antibodies (Ab): autoantibodies against endomysium (EMA) and enzyme tissue transglutaminase type 2 (tTG2), as well as anti-food Ab against deamidated peptides of gliadin (DGP), and finally by a small intestine enteropathy (1). For many years CD has been diagnosed by demonstrating of a clear small bowel enteropathy related to gluten dependence. The first guidelines of ESPGHAN (European Society of Gastroenterology, Hepatology and Pediatric Nutrition), formulated in 1970, envisaged three biopsies for diagnostic confirmation: the first on a gluten-containing diet to demonstrate the damage of the duodenal mucosa, the second after exclusion diet to verify the clear improvement of the intestinal mucosa, and the last one during re-exposure (challenge) to observe the new deterioration of the mucosa (2). The diagnostic criteria were revised in 1990, establishing that the diagnosis was based on some mandatory criteria (characteristic histological alteration of the duodenal mucosa on gluten-containing diet and unequivocal and complete clinical remission on a gluten-free diet) and also on accessory criteria as positive EMA since 1990 and anti-tTG2 since 1997 at the time of diagnosis with their normalization on a gluten-free diet and compatibility of the genetic test with the presence of the HLA alleles (3). Those criteria can still be considered valid with some exceptions evaluated in the review of the criteria carried out in 2012 by the ESPGHAN’s Working Group (1). Because of the current availability and reliability of the serological tests, the re-exposure to gluten (diagnostic challenge) is no longer considered necessary, except in some situations such as in patients with a doubtful serology.

## II. SEROLOGICAL TESTS

### Human tissue anti-transglutaminase antibodies type-2 IgA and IgG

The IgA class anti-tTG2 Ab remains the first-use serological test. The anti-tTG2 class IgA Ab represent an excellent screening procedure characterized by high sensitivity (SE) (78–100%), specificity (SP) (90–100%) and positive predictive value (PPV) (72%) and are considered the best serological screening marker for CD in individuals with normal serum IgA levels. (4,5). Despite being considered the most reliable test, even today it is very often prescribed, erroneously, in combination with other less sensitive serological markers such as anti IgG-class tTG2, with lower sensitivity, that should be limited to the few cases of serum IgA deficiency and, because of its higher costs, it should never replace the total IgA determination to exclude the immune deficiency syndrome of serum IgA. It has been well known that levels of anti-tTG2 IgA higher than 10 times the cut-off value are associated to intestinal mucosa atrophy with a high probability (1).

### Antiendomysium IgA and IgG antibodies

It is a very reliable but operator-dependent test and therefore burdened, in inexperienced hands, with a certain amount of false negative and/or false positive results with low titer. The test for EMA is highly specific, close to 100% (SE 86–100%; SP 97–100%; PPV 83%) (6), however it should only be used as a confirmatory test for anti-tTG2 IgA considering the high cost, use of animal substrate (monkey esophagus) and operator dependence (5).

### Antigliadin Deamidated antibodies IgA and IgG

IgA and IgG class anti deamidated gliadin Ab (DGP) have lower performance than anti-tTG2 and EMA and should be used exclusively in patients under three years of age (6). Anti-DGP Ab are incorrectly prescribed, quite frequently; they had been developed in 2006 as an alternative to IgA class anti-tTG2 for CD screening (7), although their performance was shown to be lower than the anti-tTG2, according to most literature data (4,8,9). Finally, the usefulness of IgG class anti-DGP should be limited to children under the age of three or in IgA deficient individuals. Determination of Anti-Gliadin Ab of 1st generation (AGA) is no longer recommended to identify individuals with CD, due to their low SE (42–100%), SP (47–94%) and PPV (18–31%). However, the AGA test is still prescribed, not understandably, in a surprisingly high percentage of cases in different regional realities (12).

### Total IgA titration

One individual per 500 of the general population has a total IgA deficiency (values <6 mg/dl, dosed with a pediatric kit, when the laboratory indicates a result <25 mg/dl). In this case IgA class tests may result falsely negative and IgG class tests are very useful especially considering that total IgA deficiency is associated with a 10 times higher risk of having CD.

### HLA typing (Haplotype Search DQ2 / DQ8)

This test has a high and almost absolute negative predictive value (close to 100%) but a low PPV and therefore its main role is to exclude (completely or almost completely) a CD diagnosis. It is useful in first-degree relatives, especially in children, to avoid redundant serological investigations by identifying subjects to be monitored periodically (every 2 years up to 6 years of life, every 4–5 years thereafter) due to the risk of developing the disease in 10–12% of cases (1). It could be helpful even in cases of doubtful interpretation.

## III. THE ROLE OF INTESTINAL BIOPSY

The accurate revision of CD diagnostic criteria achieved in the last 10 years has led to a certain loss of histology specificity due to image detection with slight damage of the duodenal mucosa, demonstrating that the enteropathy has partially lost its central role in the diagnostic approach of CD; at the same time, in the last 10 years, the importance of serology and HLA typing has increased. Because of this reason, in 2012 a new diagnostic algorithm has been proposed mostly based on genetics and Ab. However, till now, it has been prudent to correlate always the histological features with clinical manifestations, serological and genetic tests.

## IV. THE “NEW” DIAGNOSTIC CRITERIA

In 2012, the ESPGHAN’s group published new diagnostic criteria for CD with the aim of minimizing the inconvenience for patients, avoiding DB (1). The current problem is to be able to identify symptoms that could be assessed during the follow-up. It resulted that clinical suspects could be considered when referred to clear and unequivocal signs of malabsorption (celiac crisis, failure to thrive, iron anemia). It was also crucial to carefully consider the accuracy of laboratory tests used (10), better if carried out by the Reference Centers for diagnosis in order to contain costs, avoiding unnecessary waste.

The correlation between elevated Ab titers and severe intestinal mucosal damage (11) was also highlighted in the revised protocol. The proposal by ESPGHAN is aimed at pediatric patients with a clinical picture of malabsorption and symptoms related to gluten intake, in presence of:

- Serum anti-tTG2 IgA titers above 10 times upper limit of normal (10xULN) in a calibration-curve–based test;
- Positive EMA-IgA in a second blood sample
- Positive HLA-risk alleles. (HLA DQ2 and/or DQ8).

Three phenotypes of patients had been defined in the current guidelines: patients with severe malabsorption (classical form), subjects with moderate-to-low risk of celiac disease (which include non-classical and subclinical forms) and celiac family members.

Nonetheless it is common that screening diagnostic test for CD are overly prescribed also in very improbable cases, causing unjustified alarms and anxiety for the family that thus requires a “second opinion” from other specialists and/or diagnostic centers, hence increasing costs for patients and for the National Health System (NHS) (12).

The ESPGHAN’s working group is planning to publish some proposals to modify the protocol, which have also emerged from the examination of recent published series (10). A first proposal could concern the associated symptomatology: it appears quite evident that the greatest importance could be attributed to the Ab titers, regardless of the type of associated symptoms that do not necessarily have to be represented by malabsorption symptoms. Secondly: the genetic pattern would not seem to play a fundamental role in the proposal to avoid DB: in fact, if all the above criteria are met, the HLA is practically positive in 100% of the cases and therefore the test could be avoided thus realizing a significant reduction in spending of the NHS funds.

The proposal to confirm the value of the IgA anti-tTG2 Ab in a laboratory other than the first, remains with the EMA IgA test as confirmation. The doubt that remains is whether it is still necessary to maintain a double diagnosis protocol at this point, one for symptomatic patients and one for asymptomatic patients; it is probable that the latter is no longer indispensable.

## V. PREMISE AND OBJECTIVES

The aim of this study is to evaluate the new diagnostic criteria, in particular the accuracy of serologic tests and their reliability to predict CD. A second objective is to understand the impact of HLA-typing, EMA-IgA and endoscopic findings in a CD diagnosis.

## VI. PATIENTS AND METHODS

We collected data from a series of patients with diagnosis of CD performed from January 2012 to December 2018 in a tertiary Center for CD (Cava de’ Tirreni Campus of University Hospital of Salerno).

A total of 642 new celiac diagnoses were performed, 481/642 (75%) were aged less than 18 years at the time of diagnosis with mean age of 6.94 years (+4.37) and an F:M ratio of 1.8: 1.

A chart review regarding data about family history, symptoms at onset, serology tests, HLA genotype, Marsh stage of biopsy (if performed) and follow-up had been analyzed.

Patients with anti-tTG2 IgA value exceeding 10 times cut-off, according to ESPGHAN’s guidelines, received proposal of diagnosis avoiding DB, otherwise patients with values between 3–10 times underwent DB. Patients showing anti-tTG2 IgA between 1–3 times cut-off were followed quarterly and, in case of persistent altered values, DB was performed.

In the first year of application of the new ESPGHAN’s criteria (year 2012), DB was however planned for all patients regardless of clinical and laboratory findings to evaluate Positive Predictive Value (PPV) of the protocol.

The collected data were inserted in an electronic sheet(Microsoft Excel®), checked and exported for subsequent statistical analysis. The results of the study were expressed in absolute and relative frequencies for qualitative variables and mean and standard deviation (SD) or median (Md) and interquartile range (IQR) for the quantitative variables. To compare quantitative variables between groups, Pearson correlation was used. Relevant results taken in consideration were those with P<0.05. All data analysis and graphic were done in statistical software Graphpad Prism version 5.

## VII. RESULTS

**Fig. 1:**
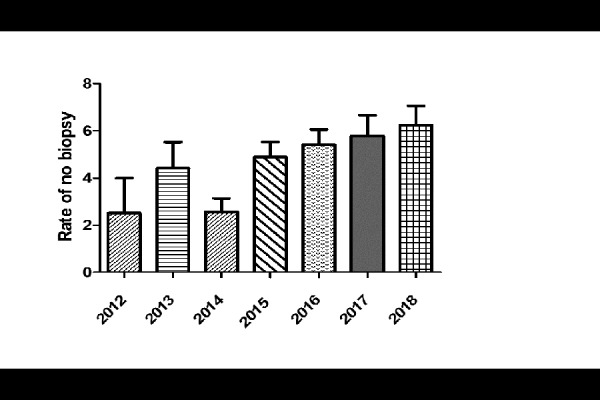
Prevalence of CD diagnosis biopsy sparing during 2012–2018 (data from 2012 regard presumptive number of patients who could had avoid DB if applied ESPGHAN’s criteria)

In the first year of application of the new ESPGHAN’s criteria (year 2012), intestinal biopsy was performed in 59 of 61 patients with a PPV of 100%. All these patients had a histological grade of Marsh type 3. After 2012, over the following years the number of biopsy-sparing patients has progressively increased (fig. 1).

**Fig. 2:**
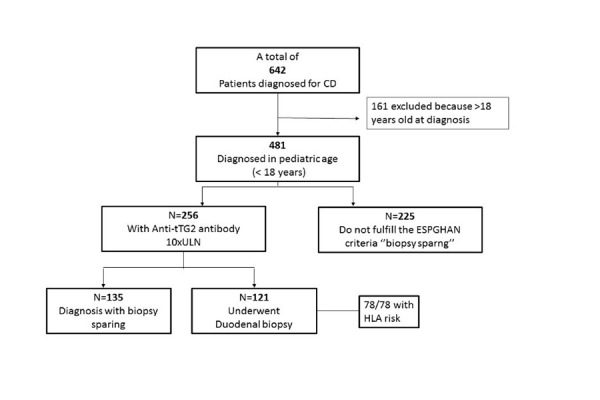
Flow chart of eligible, recruited, and excluded patients and CD diagnosis of final cohort, classified for Anti-tTG2 and biopsy

The flow-chart of final cohort is summarized in Fig. 2.Out of the overall 481 pediatric patients diagnosed for CD in our Center, a total of 256 patients presented anti-tTG2 IgA Ab titers >10xULN and 225 had Ab titers < 10xULN. A strong negative correlation was demonstrated between anti-tTG2 value and biopsy (−0.229, p < 0.001). Overall, 28% (135/481) of the patients in our series didn’t undergo DB, while 346/481 performed it and the histological degree was found to be “atrophic” (Marsh 3b or 3c) in 88% of them.

Median age of biopsy-sparing patients was 4.51 years [1–17 yrs] while median of patients that underwent to biopsy was 6.48 years [1–17 yrs]. We found a significantly positive correlation between the age and biopsy (0.213 with *p value <0.001*). On the contrary there was not significantly difference in terms of gender (−0.038 *p value >0.05*). With regards to the onset symptoms, in our cohort the 18,84% of patients was completely asymptomatic at diagnosis.

**Fig. 3:**
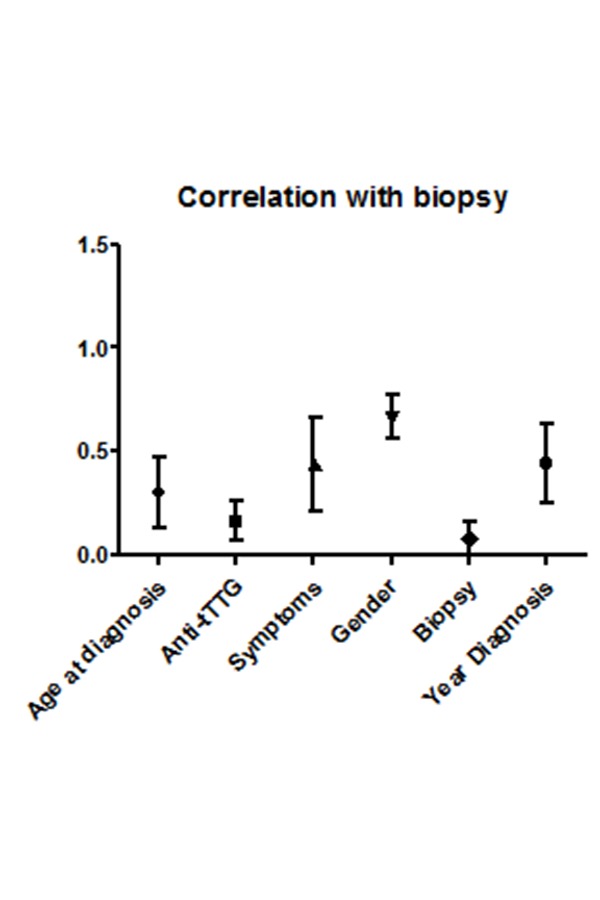
Correlation between age at diagnosis, Anti-tTG2 values, symptoms, gender and years of diagnosis with biopsy. Anti-tTgG2 and age at diagnosis were significantly correlated with biopsy

Classical onset symptoms reported were irregular alvo (28.71%), irritability (5.38%), failure to thrive (39.70%), globous abdomen (14.69%) while non-classical symptoms were asthenia (13.36%), anemia (18.96%), constipation (12.13%), isolated vomiting (8.56%), loss of appetite (21.47%) and recurrent abdominal pain (25.39%). All patients biopsy sparing were symptomatic with negative correlation between symptoms and biopsy (−0.229 with *p <0.05)* Fig 3.

The HLA risk was tested in 202/256 patients (79%) with antibody 10xULN: 163 carried the DQ2 (80.7%), 26 the DQ8 (12.9%), 12 both DQ2–DQ8 (5.9%) and 1 the DQ2–DQ7 (0.5%).Fifty-three per cent (135/256) with antibody 10xULN didn’t undergo DB and 121 however underwent DB, because of scarceness of symptoms (84/121) or discovered during familiar and territorial screening (37/121). It was largely reported I and II grade familiarity in our patients. All the 121 patients tested had a Marsh type 3 histological grade. Seventy-height/121 patients had been tested for HLA risk that was present in all 78/78 cases.

Fourteen patients were considered potential suspects at onset and were followed until diagnosis (2.9%). Mean age of these patients was 7.1 years with an F:M ratio 1.2:1. In this sub-group of patients the average level of anti-tTG2 IgA was corresponding to 4xULN, with a range from 1xULN to 6xULN. In all cases, HLA risk was present (13 DQ2, 1 DQ8).

## VIII. DISCUSSION

After the first year of application of the revised ESPGHAN’s criteria (year 2012) when the intestinal biopsy was performed in almost all patients (97%) with the declared purpose of testing their applicability, over the following years the number of patients progressively reached 79% of the total. Moreover, around 50% patients of our cohort benefited from this biopsy sparing approach, with reduced burden and risks of endoscopy and anesthesia. Since the publication of the 2012 ESPGHAN’s criteria, several authors investigated the accuracy of new criteria, both in children and adults (10, 13,14,15).

The main strength of our study is the larger retrospective cohort from single tertiary center, with the same tTG2 tests being applied through a standardized system following all manufacturers’ directions, by using the same calibration curves on automatized machines with fixed settings, involving the same laboratory technicians.

The sensitivity of anti-tTG2 IgA Ab was confirmed high and EMA improved the positive likelihood ratio and strongly correlated to diagnosis of CD.

Median age of patients who did not perform DB was lower than those that performed it; this “age-effect” is likely due to the higher relevance of onset symptoms such as malabsorption in younger children.

With regards to the onset symptoms, the classic onset symptoms were predominantly represented by irregular alvo and weight loss, whilst non-classical onset symptoms were mainly represented by anemia, inappetence and recurrent abdominal pain and it was not to be neglected the constipation present in 12% of the cases. This confirms that it is necessary to think about the diagnosis of CD even in the presence of non-usual onset symptoms.

All the patients who underwent DB, despite Ab titre>10xULN, because of a mild or completely asymptomatic clinical picture, showed however mucosal atrophy.

We also stress that the application of diagnostic criteria offers the possibility to avoid DB but this represents an option, not a mandatory rule, that must be discussed with the child’s parents, in the presence of a diagnosis that involves the adoption of a restrictive “quod vitam” diet. For this reason, they could request to the gastroenterologist to carry out in any case the histological examination of the duodenal mucosa in order to reach an absolute diagnostic certainty.

Moreover, our data revealed that HLA-typing for DQ2/DQ8 does not improve accuracy of CD diagnosis without biopsies and can be omitted for this purpose, with 100% of HLA-risk alleles in patients with anti-tTG2 IgA ≥10xULN and positive EMA.

The limitation is the retrospective nature of our study, as we considered only patients with diagnosis of CD and potential disease without other differential diagnosis, that could represent a bias in our cohort.

In our cohort only 2.9% of potential celiacs were diagnosed. In this group the average level of anti-tTG2 IgA was about 4xULN, ranging from 1x to 6x. In no case did the Ab titer exceed 6x the limit with EMA IgA present in all cases and all were HLA positive. In our Center, over the last years, a new line of conduct has been established that foresees a vigilant waiting in cases of anti-tTG2 IgA < 3xULN with accurate follow-up and this has certainly determined a lower incidence of diagnosis of potential CD avoiding to subject to DB the patients with very low Ab titers. This strategy makes it possible to select the group of potential CD that tends to maintain positive serology over the years and that, often after some time, develops the atrophy of the intestinal mucosa.

## IX. CONCLUSIONS

This study confirms that the ESPGHAN biopsy-sparing approach allows a correct diagnosis of CD while saving costs for health care systems as well as discomfort for the patients.

Nonetheless some adjustments are needed in analyzing our experience in the current study.

An important proposal is to modify the current guidelines concerning clinical scenarios: it is quite clear that the greatest importance is to be attributed predominantly to the Ab titer > 10xULN, regardless of the type of associated symptoms that do not necessarily have to be represented by malabsorption symptoms or others “classical” symptoms.

An additional point regards genetic pattern that does not seem to play a fundamental role in the proposal to avoid DB: in fact, if the aforementioned criteria are met, HLA is positive in 100% of the cases and therefore the test could be avoided, thus realizing an important reduction in spending for the health care systems.
